# Comparative Analysis of Microbial Diversity Across Temperature Gradients in Hot Springs From Yellowstone and Iceland

**DOI:** 10.3389/fmicb.2020.01625

**Published:** 2020-07-14

**Authors:** Peter T. Podar, Zamin Yang, Snædís H. Björnsdóttir, Mircea Podar

**Affiliations:** ^1^Biosciences Division, Oak Ridge National Laboratory, Oak Ridge, TN, United States; ^2^Faculty of Life and Environmental Sciences, University of Iceland, Reykjavik, Iceland

**Keywords:** hot springs, thermophiles, microbial ecology, rRNA amplicons, biogeography

## Abstract

Geothermal hot springs are a natural setting to study microbial adaptation to a wide range of temperatures reaching up to boiling. Temperature gradients lead to distinct microbial communities that inhabit their optimum niches. We sampled three alkaline, high temperature (80–100°C) hot springs in Yellowstone and Iceland that had cooling outflows and whose microbial communities had not been studied previously. The microbial composition in sediments and mats was determined by DNA sequencing of rRNA gene amplicons. Over three dozen phyla of Archaea and Bacteria were identified, representing over 1700 distinct organisms. We observed a significant non-linear reduction in the number of microbial taxa as the temperature increased from warm (38°C) to boiling. At high taxonomic levels, the community structure was similar between the Yellowstone and Iceland hot springs. We identified potential endemism at the genus level, especially in thermophilic phototrophs, which may have been potentially driven by distinct environmental conditions and dispersal limitations.

## Introduction

Archaea and Bacteria inhabit nearly every environment on Earth, including many that are inhospitable to multicellular life, such as hot springs. As geothermal water cools, outflowing from the source, temperature, chemical and redox gradients form. Distinct microbial communities occupy the various niches of such gradients, based on individual species adaptation to different temperature and chemical optima (Stetter, 1999; Reysenbach and Shock, 2002). Hot springs around the world have been used for decades as natural laboratories to study the effect of environmental parameters on microbial evolution, diversity and physiology (Castenholz, 1969; Brock and Darland, 1970; Skirnisdottir et al., 2000; Purcell et al., 2007; Hamilton et al., 2012; Menzel et al., 2015; Alcorta et al., 2018). Extreme temperature and pH values have been shown to have the largest contribution in restricting microbial diversity, although the magnitude of their effects are dependent on the hot spring and were also influenced by geochemistry and other environmental factors (Meyer-Dombard et al., 2011; Hamilton et al., 2012; Cole et al., 2013; Wang et al., 2013; Sharp et al., 2014; Chiriac et al., 2017; Merkel et al., 2017; Power et al., 2018; Tang et al., 2018; Zhang et al., 2018).

Hot springs have also been used to test hypotheses on factors and mechanisms that lead to microbial diversification and biodiversity patterns (Ward and Cohan, 2005; Martiny et al., 2006). The hypothesis that microbes in general have a high dispersal rate, that would homogenize genetic variations that may arise as result of local ecological and evolutionary events, has been challenged by studies of microbes in geothermal hot springs (Whitaker et al., 2003; Papke and Ward, 2004). Because numerous thermophilic microbes that do not form endospores do not survive for extended periods desiccated in air (Castenholz, 1969; Beblo et al., 2009) their dispersal ability over large geographic distances is limited. Therefore, while microbial communities that inhabit geochemically similar hot springs on different continents are expected to be physiologically and taxonomically similar, some of the individual species may evolve as endemic populations, similar to plants and animals on distant islands. This has been demonstrated by comparing thermophilic *Synechococcus* (Bacteria) and *Sulfolobus* (Archaea) in hot springs from North America, Europe and Asia (Papke et al., 2003; Whitaker et al., 2003; Becraft et al., 2020).

Here we studied the microbial diversity across temperature gradients in three alkaline hot springs from Yellowstone National Park (YNP) and Iceland for which there was no prior microbial data available. We hypothesized that even though those individual hot springs are geographically isolated, they would share the same general microbial community composition at high taxonomic levels (and potentially physiological activities) at similar temperatures along the gradient. While multiple previous studies identified clear effects of temperature on community richness (Cole et al., 2013; Cuecas et al., 2014; Sharp et al., 2014; Power et al., 2018) some studies on springs with temperatures below 80°C did not (Wang et al., 2013). Selecting hot spring gradients that span a wide range of temperatures up to boiling, enabled us to test the degree of diversity variation across temperature intervals. At the same time, because of the large geographical distance separating the hot springs in North America from those in Iceland, we performed analyses for potential genetic variation between shared taxa at equivalent temperatures. As relatively few comparative studies of distant hot springs around the world have previously been conducted, we aimed to expand such microbial diversity comparisons and also enable future integrative studies.

## Materials and Methods

### Icelandic Hot Spring Samples

**FIGURE 1:**
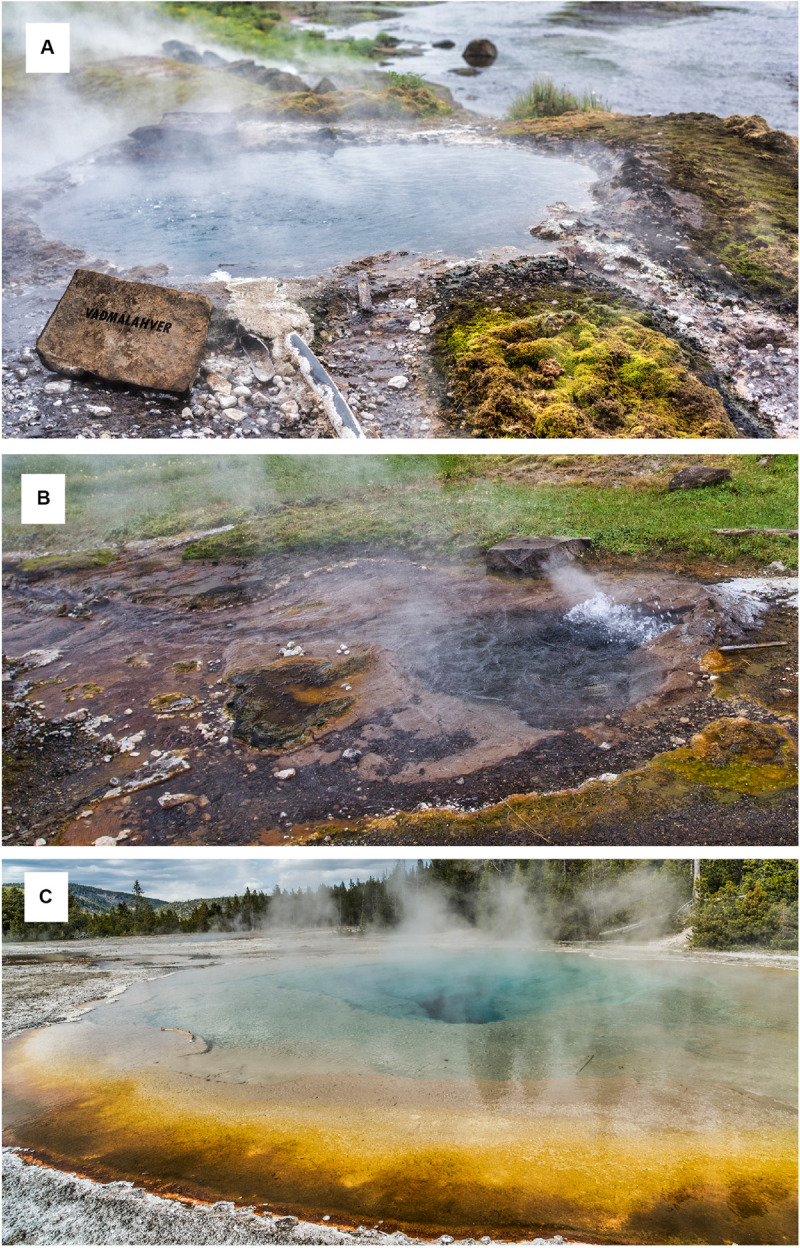
Overview of sampled thermal features. **(A)** Vaðmálahver, Flúðir (Iceland). **(B)** Hurðarbak, Borgarfjörður (Iceland). **(C)** Mirror Pool, Yellowstone National Park (United States). Actual sample collection points are presented in the Supplementary Figures S1–S3.

Microbial mats, sediments, and water samples were collected on June 9, 2016 at a hot springs field in the village of Flúðir, Iceland (GPS coordinates 64°08′13″ N 20°18′34″ W). The main hot spring, Vaðmálahver (Figure 1), is alkaline (pH ∼8.5) and the source water is 98°C (boiling). The outflow of Vaðmálahver gradually cools and the water discharges in a nearby river. Several hot spring sources from the same site discharge in Hverahólmi, the oldest public swimming lagoon in Iceland. The temperature in the main source as well as in the outflow, sediments and in the microbial mats was measured using a Fisherbrand Traceable Waterproof Thermometer (Fisher Scientific cat no. 02-402-0) that had a stainless steel temperature probe at the end of a long (10 ft) wire cable positioned either manually or with a telescopic pole. The pH was measured onsite using non-bleeding pH indicator strips (pH 5–10 range, EMD Millipore) on source water and along the outflow. Water and sediment gravel (approximately 90% water-10% gravel by volume) from the main source were collected for geochemical analysis using a stainless-steel cup (500 ml) at the end of a telescopic pole and immediately poured into sterile 100 mL Pyrex glass bottles, capped with no air headspace and secured using butyl rubber stoppers and aluminum crimps. The samples were left to cool naturally to room temperature and then stored and transported cold. A sulfide test done on site using lead acetate strips (Sigma-Aldrich) was negative (limit of detection 3–5 mg/l). Submerged microbial mats and sediments (∼1–2 grams) were collected using sterile syringes and stainless-steel spatulas and placed into plastic tubes containing ceramic beads and 750 μl Xpedition^TM^ Lysis/Stabilization Solution (Zymo Research, Irvine, CA) and lysed by bead-beating for one minute with a battery-operated tube shaker. That ensured cellular lysis, inactivation of degradative enzymes and stabilization of the DNA until further processing. A total of seven different spots were sampled from and around the Vaðmálahver spring, ranging from 98 to 47°C (Supplementary Figure S1 and Table 1). The samples in the outflow were sequentially collected going upstream to higher temperatures, all the way to the source. This is an important consideration in sampling hot spring outflows, as collecting going downstream in the runoff would lead to contamination of lower temperature samples with sediments and mats disturbed upward. The main source of an adjacent spring (temperature of 92°C) that flows into Hverahólmi was also collected, as well as mat and water samples from the lagoon (38°C). The microbial community from the lagoon (250 ml water sample) was collected on a Millipore Sterivex 0.2 mm syringe filter and preserved by adding Xpedition solution into the filter cartridge and then capping. A microbial mat sample was collected from a submerged rock in the lagoon and processed as were the other mat samples. With the exception of the lagoon water (planktonic sample), all other samples that we collected for microbiological characterization from all the hot springs were mats and gravel/sediment. After reaching the laboratory the lysed samples were stored at −20°C until DNA extraction or at 4°C (samples for geochemical analyses).

**TABLE 1:** Environmental samples used in the study. Except for FB2.Pk, which was a water sample, all others were mats/sediment.

SampleID	Location	Thermal Feature	Temperature, °C	Replicate
MP1A	YNP	MirrorPool	52	1
MP1B	YNP	MirrorPool	52	2
MP1C	YNP	MirrorPool	52	3
MP2A	YNP	MirrorPool	58.6	1
MP2B	YNP	MirrorPool	58.6	2
MP2C	YNP	MirrorPool	58.6	3
MP3A	YNP	MirrorPool	65.5	1
MP3B	YNP	MirrorPool	65.5	2
MP3C	YNP	MirrorPool	65.5	3
MP4A	YNP	MirrorPool	69	1
MP4B	YNP	MirrorPool	69	2
MP4C	YNP	MirrorPool	69	3
MP5A	YNP	MirrorPool	72.5	1
MP5B	YNP	MirrorPool	72.5	2
MP5C	YNP	MirrorPool	72.5	3
MP6A	YNP	MirrorPool	78	1
MP6B*	YNP	MirrorPool	78	2
MP6C	YNP	MirrorPool	78	3
MP6D	YNP	MirrorPool	83	1
FV2	Iceland	Vaðmálahver	87	1
FB1	Iceland	Hverahólmi sr.	92	1
FB2.Pk	Iceland	Hverahólmi	38	1
FB3.Mat	Iceland	Hverahólmi	38	1
FV1	Iceland	Vaðmálahver	98	1
FV3	Iceland	Vaðmálahver	72	1
FV4	Iceland	Vaðmálahver	68	1
FV5	Iceland	Vaðmálahver	67	1
FV6	Iceland	Vaðmálahver	63	1
FV7	Iceland	Vaðmálahver	47	1
HD1	Iceland	Hurðarbak	100	1
HD2	Iceland	Hurðarbak	81	1
HD3	Iceland	Hurðarbak	67	1
HD4	Iceland	Hurðarbak	61.4	1
HD5	Iceland	Hurðarbak	45.5	1

*MP6B^∗^ did not yield sufficient sequences.*

The second location in Iceland was also an alkaline spring (pH 8.0) at Hurðarbak (Figure 1), in the Borgarfjörður region [GPS coordinates 64°41′18″ N, 21°24′10″ W], approximately 80 km NW of Flúðir. Samples were collected on June 16, 2016. Five sampling spots were selected, with temperatures ranging from 99–102°C (the source spring) to 46°C in the outflow channel (Supplementary Figure S2 and Table 1). For geochemical analysis a water sample was collected from the source spring. Sample collection and processing were performed as described above.

### Yellowstone National Park Hot Spring Samples

Microbial mats, sediment, and water samples were collected on December 31, 2016 at Mirror Pool, an alkaline (pH 8.0) thermal feature from the Upper Geyser Basin, in the Cascades Group [GPS coordinates 44°28′59″ N, 110°51′01″ W] (Figure 1). Nine sites were sampled along the main pool and gradient outflow of the spring, where temperatures ranged between 83°C–52°C (Supplementary Figure S3 and Table 1). Because the outflow of the pool was larger than for the hot springs in Iceland it was feasible to identify spots with the same temperature accessible for collection using spatulas. Therefore, three adjacent replicate samples (∼1 cm^3^ each) separated by less than 10 centimeters were collected for each temperature value, to determine the degree of diversity fluctuation across small scales. From the main pool, the collected samples were more distant and were scooped out from the stainless-steel canister. A water sample for geochemical analysis was also collected from the main pool. The samples were collected and processed as already described.

### Water Chemistry Analysis

The chemical composition of the hot spring source water samples was performed at The University of Tennessee Knoxville Water Quality Core Facility. Carbon and nitrogen content were determined by thermal combustion and infrared detection with a Shimadzu carbon/nitrogen analyzer. The concentration of metals was measured by inductively coupled argon plasma (ICP) optical emission spectrometry using a Thermo-Scientific iCAP 7400 ICP spectrometer. Ions were measured by ion chromatography with a Thermo-Scientific^®^/Dionex ICS-2100 (anions) and ICS-1100 (cations), with background suppression for low detection limits.

### DNA Extraction

Total genomic DNA from environmental samples was isolated using the ZR Soil Microbe DNA Kit (Zymo Research) following the manufacturer’s protocol. To isolate DNA from the high temperature sediment samples collected in the larger volume bottles, 25 mL of those samples subjected to centrifugation (12,000 × *g* for 20 min), the water carefully decanted, and the pellet suspended and lysed using the Zymo Lysis/Stabilization Solution following by processing as above. The concentration of DNA was determined using a Qubit dsDNA HS assay kit and fluorometer (Thermo Fisher Scientific).

### Microbial SSU rRNA Gene Amplicon Sequencing

The V4 hypervariable region of the small subunit ribosomal RNA gene (SSU rRNA) was amplified using universal bacterial/archaeal 515F and 806R primers (Bates et al., 2011) fused to Illumina sequencing adapters, following the procedure developed by Lundberg et al. (Lundberg et al., 2013). To increase the coverage of archaeal groups not recognized effectively by the standard 515F and modified 806R primers (5′ GTGCCAGCMGCCGCGGTAA and 5′ GGACTACHVGGGTWTCTAA, respectively), we supplemented the reaction with further modified versions that included 20% 515FCren (5′ GTGKCAGCMGCCGCGGT AA, for Crenarchaeota), 5% 515FNano (5′ GTGGCAGYCG CCRCGGKAA, for Nanoarchaeota) and 5% 805RNano (5′ GGAMTACHGGGGTCTCTAAT, for Nanoarchaeota), similar to what was described in Liang et al. (2018). 12-nucleotide barcode sequences were incorporated into the second stage amplification reaction to enable sample multiplexing. The final amplicons were pooled, purified using Agencourt AMPure XP bead and quantified using Qubit. A diluted purified pooled amplicon sample (9 pM), containing 20% phiX DNA was denatured and sequenced (2 × 250 nt) on an Illumina MiSeq instrument (Illumina Inc., San Diego, CA) using a v2 500 cycle kit, according to manufacturer’s protocol.

### Amplicon Sequence Analyses

The amplicon primer regions were trimmed from the raw FASTQ sequence files using cutadapt (Martin, 2016). The sequence reads were then de-multiplexed based on barcode sequences using the QIIME (Caporaso et al., 2010) python script split_libraries_fastq.py followed by splitting by individual samples using split_sequence_file_on_sample_ids.py. For one of the samples (MP6B) the number of sequences was very low (<500 sequences) and that sample was removed from analyses. Demultiplexed FASTQ paired reads were imported into QIIME2 v2019.7 (Bolyen et al., 2019) on a desktop computer. The reads were paired with VSEARCH (Rognes et al., 2016), quality filtered and denoised using Deblur (Amir et al., 2017). Resulting amplicon sequence variants (ASV) were aligned and used to generate a phylogenetic tree using the align-to-tree-mafft-fasttree pipeline from the q2-phylogeny plugin. To calculate alpha−diversity metrics [observed OTUs, Pielou’s eveness, Shannon’s index and Faith’s Phylogenetic Diversity (Faith, 1992)] beta diversity metrics [weighted UniFrac (Lozupone et al., 2007) and Bray−Curtis dissimilarity], and input the resulting matrices into principle coordinate analyses (PCoA) and visualization plots, we used the q2−diversity workflow, with rarefaction to 5000 sequences per sample (based on plateauing of the observed diversity and retaining of all samples). A general temperature classification of samples was generated by assigning each sample to groups separated by 5^o^C (from 40^o^C to 100^o^C). Environmental parameters that could impact alpha diversity were tested using Spearman correlation and analysis of variance (ANOVA), using q2 diversity alpha-correlation and q2 longitudinal (Bokulich et al., 2018b). To test for factors that contribute to microbial diversity differences between the samples (actual temperature, general temperature, location, hot spring) we used multi-way permutational multivariate analysis of variance (PERMANOVA) (q2 diversity adonis tests), comparing the variance explained by the various parameters singly or in combinations. Pairwise tests within metadata categories were performed by one-way PERMANOVA using the q2 diversity beta-group-significance.

To assign taxonomy to ASVs we used the q2−feature−classifier (Bokulich et al., 2018a) (classify−sklearn) against the Silva-132-99 SSU rRNA database (Pruesse et al., 2007). A table with the taxonomic classification of the reads for every sample is provided as a supplementary file (Supplementary Table S1). The raw FASTQ files are available in the NCBI SRA (accession numbers SRR11066910–SRR11066942, Bioproject PRJNA605860).

### Phylogenetic Analyses

Phylogenetic trees to compare selected Yellowstone and Iceland ASVs with related organisms from GenBank were generated using PhyML in the software package Geneious^1^. BLASTN search algorithm was used to identify relatives of the Yellowstone and Iceland bacteria and archaea in public sequence databases followed by the phylogenetic reconstructions.

## Results and Discussion

### Geochemical Comparisons of the Three Hot Springs

The three hot springs were selected because of their high temperature (80–100°C at the source), similarities in pH and the presence of discharge channels with gradual cooling that harbor distinct microbial mats. Mirror Pool is a large (∼15 × 20 meters) non-erupting deep pool, in the Upper Geyser Basin thermal region (Cascade Group complex) of Yellowstone National Park. Abundant silica deposits are present both in the pool and on its edges and the outflow channel. The temperature and pH we recorded are similar to those reported in the YNP Research Coordination Network database^2^, 76–80°C and pH 8, measured in 1999 although we could not find previous geochemical data. Similar to other alkaline-siliceous chloride-type springs in that thermal region (Fournier, 1989), Mirror Pool has high levels of chloride, sodium, silica and arsenic but is low on sulfur or sulfate, calcium and magnesium (Table 2). While the concentration of dissolved sulfide was below the limit of detection using lead acetate test strips (∼5 mg/l), other hot springs in the Cascade Group, which share the same overall chemistry, were shown to be very low in sulfide (0.02 mg/l) (Thompson and DeMonge, 1996). Its relatively low flow rate and close proximity to the forest line are probably linked to the relatively high dissolved organic carbon (∼100 mg/l). Unlike Mirror Pool, the two sampled hot springs in Iceland were much smaller (<2 m in diameter), boiling and actively discharging, likely explaining their lower dissolved organic carbon content (10–20 mg/l). The overall mineral content of both springs was also lower than that of Mirror Pool, although they had higher levels of sulfate, calcium, iron and sulfide. Sulfide concentration is also higher in the two Icelandic hot springs (1.34 mg/l in Vaðmálahver and 1.2 mg/l at Hurðarbak), based on published measurements (Arnorsson and Gunnlaugsson, 1983; Ali, 1997). We could not find matched data in the literature on other dissolved gasses that we could not measure onsite (O_2_, H_2_, CO_2_, CO, CH_4_). We recognize therefore that, as the water flows from the source and cools, there may be changes in the water chemistry that we have not accounted for, such as dissolved gases, precipitation of minerals, microbial metabolic products.

**TABLE 2:** Chemical composition of hot spring water samples.

	Mirror Pool	Vaðmálahver	Hurðarbak
TC	101.6	24.39	11.94
TIC	4.84	3.01	2.24
TOC	96.79	21.38	9.71
Cl	271.79	24.89	32.01
NO3	0.19	0.06	0.07
SO4	13.78	54.97	57.13
HPO4	0.1	0.13	0.15
F	19.34	1.14	1.84
Na	400.09	78.23	69.87
K	10.3	0.73	0.83
Mg	0.02	0.11	0.08
Ca	0.75	5.89	4.26
Al	0.33	0.05	0.05
Cu	0	0	0.01
Fe	0.04	0.15	0.17
Si	119.55	31.38	20.23
S	4.69	25.03	23.01
Ni	0.03	0.01	0
Pb	0.18	0.04	0.03
Cr	0.01	0.01	0.01
Be	0.01	0	0
As	1.32	0.01	0.02
Sb	0.03	0	0
Y	0.01	0.01	0.01

*All values in mg/l. TC, total carbon; TIC, total inorganic carbon; TOC, total organic carbon. Mn, Zn, Cd, Co, Ba, Se, Tl, Ag: not detected (<0.01 mg/l).*

### Temperature Differentially Influences the Microbial Alpha Diversity

The combined sequencing of the SSU rRNA amplicons from all samples resulted in over 5.7 million sequences. After de-multiplexing, quality-based filtering, denoising, chimera and singleton removal, the number of sequences for individual samples ranged from 5,315 to 332,920, with an average of ∼115,000 sequences per sample and a total of 1729 amplicon sequence variants (ASVs) (unique taxa). Because the type of clustering algorithm and selection of similarity level impacts the number of traditional operational taxonomic units (OTUs), we only used ASVs for calculation of diversity indices. Depending on the degree of sequence variability in the V4 region for the various taxa, we expect ASVs to provide resolution to genera, species and below, based on comparisons of know species of Archaea and Bacteria [e.g (Shakya et al., 2013)].

**FIGURE 2:**
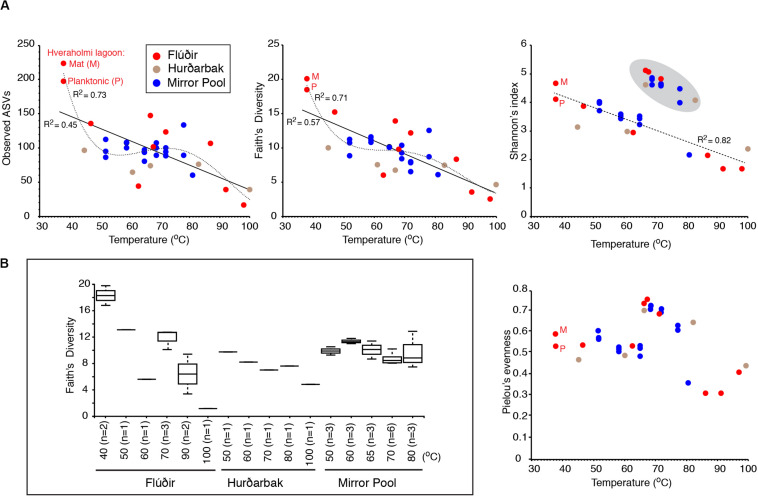
Microbial alpha diversity versus temperature. **(A)** Scatterplots of various diversity indices versus temperature. Linear or polynomial regression and goodness of fit are shown. For the Shannon’s index, the effect of excluding the upper cluster values (circled) on linear regression fit is shown. **(B)** Scatterplots of diversity indices vs. temperature after excluding the Cyanobacteria and Chloroflexi sequences.

For studying the microbial diversity within each sample (alpha diversity) we used both direct counts of the number of ASVs as well as metrics that take into account the evenness of diversity (Pielou’s evenness), abundance and distribution of the taxa (Shannon’s index) or the phylogenetic diversity (Faith’s diversity). In Mirror Pool, where we were able to take spatially separated samples at the same temperature, there were differences in alpha diversity between environmental replicates, with the coefficient of variation ranging from 0.2%–12%. The higher deviations were for high temperature samples. Those differences were, however, minor compared to the temperature-linked differences. At the Icelandic hot springs, temperature had the largest impact and was inversely correlated with the number of detected taxa (ASVs) and phylogenetic diversity (Spearman *p* = 0.006 and *p* = 0.000, respectively) (Figure 2). The decline in number and diversity of microbial taxa with temperature does not appear to be linear and is steeper in the ranges corresponding to the transition between mesophily and thermophily (35–45^o^C) and between thermophily and hyperthermophily (>80°C). Such non-linear relationships have been previously reported for hot spring communities in Canada, New Zealand, United States (Nevada) and Thailand where wide temperature ranges were present within individual thermal systems (Cole et al., 2013; Cuecas et al., 2014; Sharp et al., 2014). ANOVA tests of potential multiple effects on the alpha diversity confirmed that, while the temperature value had the largest influence (*P*-Value from F-Ratio = 3.4e-05), the individual thermal feature was significant too (*P*-Value from F-Ratio = 3.6e-03, passing pairwise T-tests with BH-FDR). In Mirror Pool, where the available temperature range was narrower, its effect on alpha diversity was minor. When only strict thermophilic temperature values were analyzed for the Icelandic features as well (50–80°C, the range sampled in Mirror Pool), the temperature effect on alpha diversity was absent, which may explain reports of no temperature effect on species richness (Wang et al., 2013). Also, when analyzing the temperature distribution of non-phylogenetic alpha diversity evenness indices (Pielou’s, Shannon’s), we observed that samples spanning the 67–80°C were sharply higher relative to what appears to be relatively linear distribution across the other two temperature ranges (Figure 2). While we cannot provide a definitive explanation for these differences, one possibility is that in the 67–80°C range there are major shifts in the microbial communities, with the stratified microbial mats dominated by photosynthetic groups (Cyanobacteria, Chloroflexi) being replaced by various extreme thermophilic taxa (Thermi, Aquificae, Crenarchaeota) that are not spatially organized, with no dominating members. As the temperature further increases, the number of organisms that can survive decreases and certain taxa dominate (*Pyrobaculum, Ignisphaera, Thermocrinis*), leading to a reduced evenness. We further investigated this by calculating the Shannon’s index at different sequence similarity clustering levels (between 97% and 75% similarity levels). Interestingly, the effect is maintained even at distances corresponding to family order levels OTUs (∼90% level OTUs), and the diversity index begins to somewhat flatten for the thermophilic range in the class-phylum categories (80-75% level OTUs) but is followed by the abrupt drop at the extreme temperatures (>80°C). This suggests that the non-linear distribution of Shannon’s evenness index is linked to major shifts on how the communities are structured across the temperature gradient. These results and observations highlight the importance of sampling multiple temperature ranges in hot springs and the choice of diversity indices in studying such environments.

### Temperature and Biogeography Effects on Beta Diversity

**FIGURE 3:**
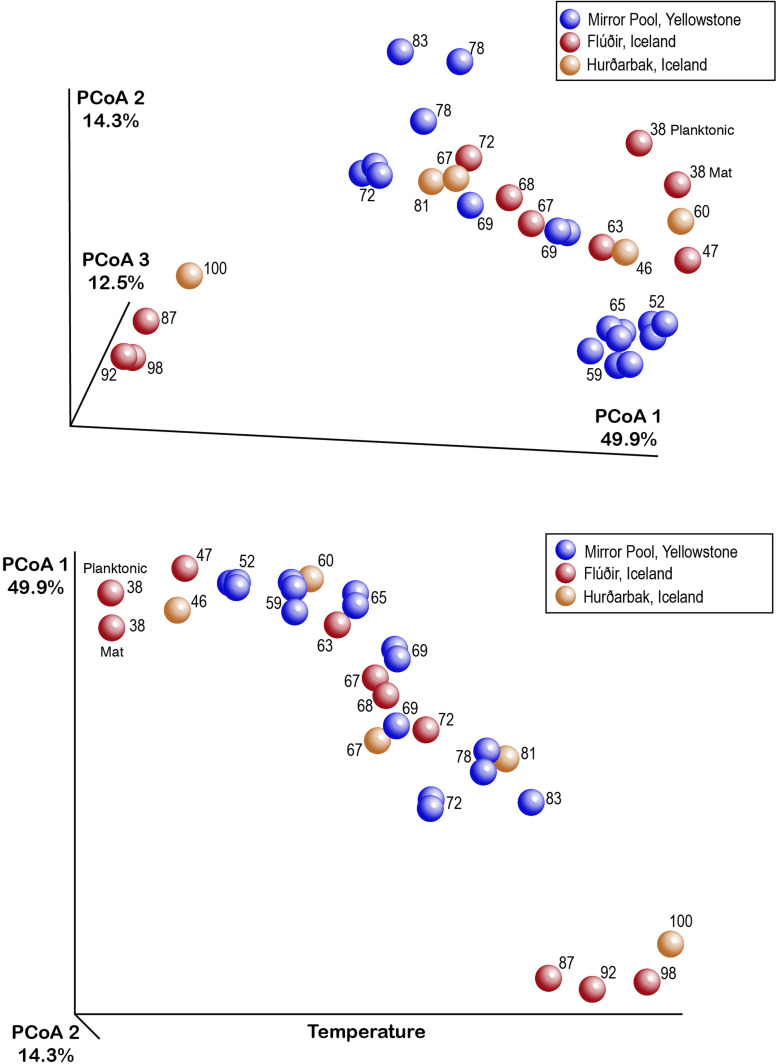
Representation of hot springs microbial beta diversity through EMPeror plots of the principal coordinates analysis output for weighted UniFrac distances.

**FIGURE 4:**
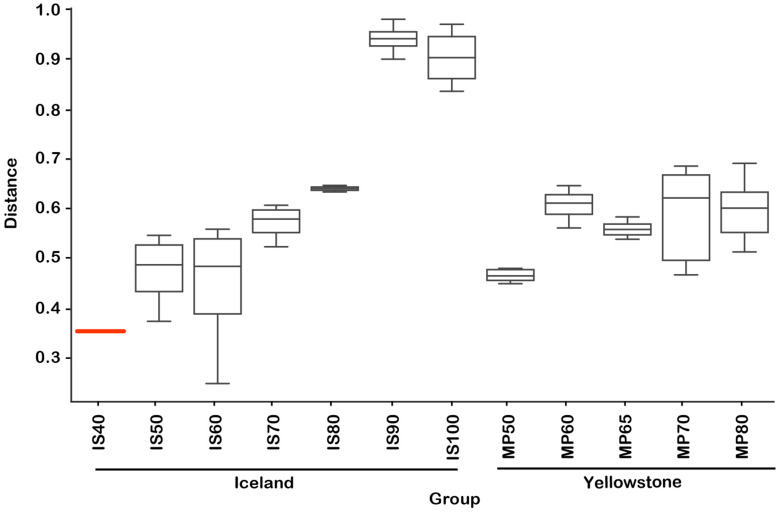
Weighted UniFrac location-temperature group significance plot. Distances are relative to the lowest temperature group (Flúðir Hverahólmi, Iceland, 40°C). PERMANOVA *F*-test significance *p* = 0.001. IS, Iceland.

While alpha diversity analyses revealed the microbial community structure within individual samples collected across temperatures and locations, it does not enable a direct comparison of communities between samples (beta diversity) (Lozupone and Knight, 2008). Because some individual ASVs may represent closely related ecotypes, species or genera, we also aimed to compare the community structure across samples, temperature and locations taking into account taxonomic (and potential physiological) relatedness of the various organisms. We therefore used weighted UniFrac as a quantitative beta diversity metric that incorporates both the phylogenetic distance between ASVs and their relative abundance (Lozupone and Knight, 2005, 2008). The multi-dimensional UniFrac distance matrices were condensed through a principal coordinate analysis (PCoA) into a three-dimensional space, in which the microbial diversity characteristics of each sample is represented by a discrete data point. Communities that have similar types and abundance of species are closer to each other in that space than those that consist of different types of microbes. The PCoA plot shows that most samples from close temperature values cluster together (are similar) regardless of the hot spring or location (Iceland or Yellowstone) (Figure 3), with most of the variation (∼77%) explained in the three main coordinates. A test of the combined effect of temperature and location (which includes underlying chemical differences) to beta diversity revealed over 80% of the variation explained by those two factors (ADONIS R^2^ = 0.877, *p* = 0.001). Temperature was, however, the most important driving factor (PERMANOVA pseudoF = 10.4, *p* = 0.001, ANOSIM *R* = 0.77, *p* = 0.001) (Figure 4), which is also evident based on the PCoA plot (Figure 3), where samples are distributed primarily by temperature rather than location or thermal feature. Most sample replicates from Yellowstone grouped tightly together, except for some at higher temperatures that display a larger dissimilarity. Therefore, even though there are distinct differences in some of the measured chemical composition of the springs at the source, these appear to be secondary factors in shaping of the microbial communities when we compared these alkaline hot springs. Shaping of the microbial communities by other physical and chemical factors have nevertheless been documented in comparisons of thermal environments, for example hydrogen concentration (Spear et al., 2005) pH (Power et al., 2018; Colman et al., 2019) and minerals (Mathur et al., 2007). We also recognize that, even though temperature was the dominant factor measured here, changes in temperature along the outflows may be driving specific changes in water chemistry that we did not measure. Such temperature-associated changes also could impact the microbial communities, but we cannot distinguish them here. For example, it has been shown that in acidic geothermal springs, water cooling results in precipitation of metals, changes in dissolved gasses, generating chemical energy gradients that in turn shape the microbial communities along the gradient (Macur et al., 2004). To our knowledge, such chemical gradients have not been measured in thermal alkaline outflows.

### Microbial Taxonomy Across Temperature Gradients and Hot Springs

**FIGURE 5:**
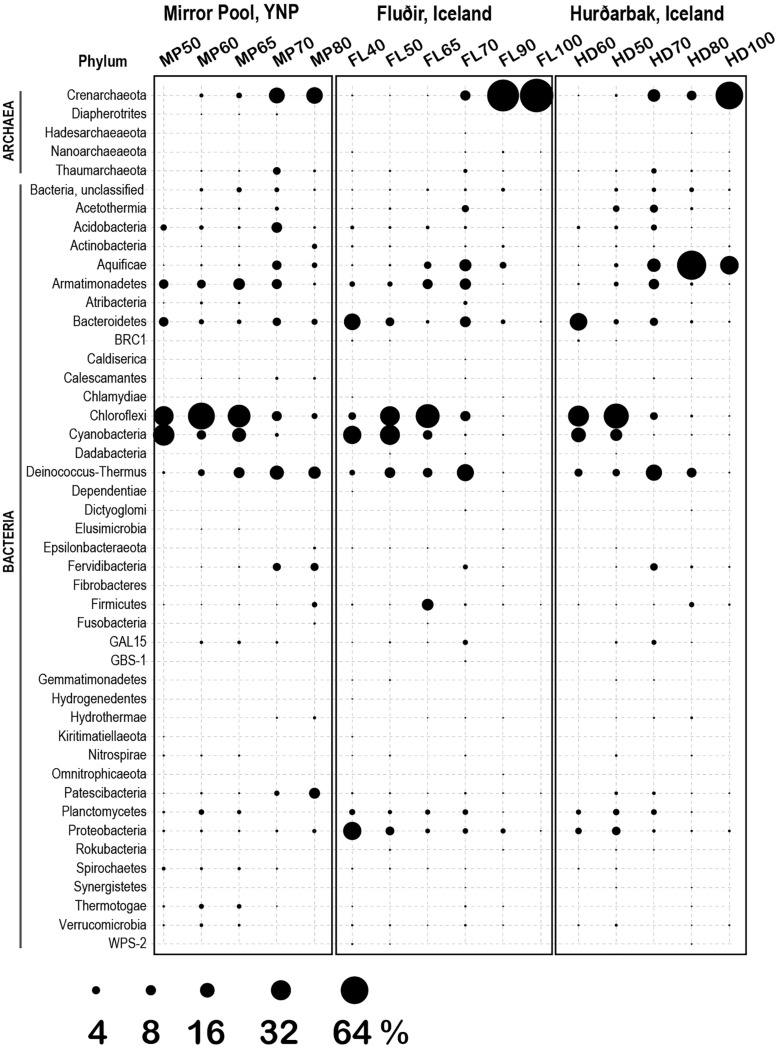
Microbial diversity (phylum level) at the three thermal springs depending on general temperature (40–100°C). Circle size indicates the inferred relative abundance, based on amplicon data (in %).

**FIGURE 6:**
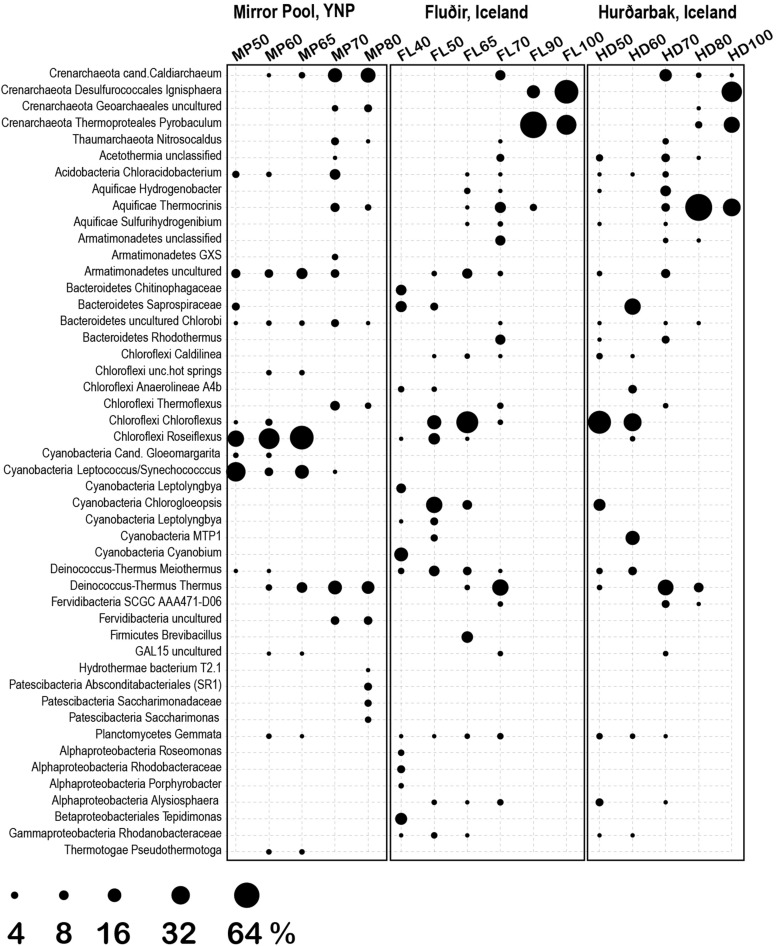
Most abundant genera/families at the three thermal springs depending on general temperature. Circle size indicates the inferred relative abundance based on amplicon data (in %).

The 1729 unique sequence variants were assigned to 182 genera, corresponding to 5 phyla of Archaea (9 classes) and 40 phyla of Bacteria (86 classes) (Figures 5, 6). The temperature gradient that forms in the three distinct hot spring systems creates distinct niches where organisms that are best adapted to those conditions thrive. In all those systems, such niches can be distinguished even macroscopically, based on the morphology and color of the mats (Supplementary Figures S1–S3). The deep amplicon sequencing that we achieved revealed the presence and relative abundance of numerous groups of organisms including the rare taxa. At the lowest temperature (38°C), the water and microbial mats of the Hverahólmi lagoon are dominated by a large diversity of mesophilic and mildly thermophilic heterotrophic as well as photosynthetic bacteria, including Alpha and Betaproteobacteria (*Roseomonas, Rhodobacter, Tepidimonas*), Bacteroidetes (*Chitinophaga, Saprospira*) and Cyanobacteria (*Cyanobium, Leptolyngbya*) (Figure 6). The overall diversity is slightly higher in the mat than in the water column although the overall community structure and taxonomic composition are the same (Figures 2, 3). Some differences may, however, be due to the difference in collection and processing between the two sample types (filtration versus complete mat biomass). As the lagoon receives a constant stream of high temperature hot spring water, we also detected numerous extreme thermophilic and hyperthermophilic archaea and bacteria in the lagoon (e.g., *Pyrobaculum*, Aquificae, Thermi) at <0.1% of total sequences. While those organisms increase the alpha diversity, the lagoon being the most diverse of the sampled niches, at tens of degrees below their optimum they most likely represent a physiologically inactive, non-dividing component of the community (Stetter, 2006). Some, depending on their capacity to survive low temperatures and exposure to oxygen, may have the potential to colonize other hot springs by different dispersal mechanisms (water, wind).

The thermal areas (50–80°C) of all three hot springs share a variety of common organisms including Armatimonadetes, Chlorobi, Planctomycetes at the lower range, and Thaumarchaeota, Aquificae (*Thermocrinis*) and Thermi, at the upper temperature range. There are though some specific differences, including in the distribution of Aigarchaeota (Crenarchaeota). In Mirror Pool we observed two distinct types of Aigarchaeota. The most abundant phylotype is related to *Candidatus* Caldiarchaeaum subterraneum, an uncultured, likely heterotrophic archaeon identified in subsurface geothermal aquifers (Nunoura et al., 2011; Takami et al., 2015). The Mirror Pool lineage was also identified in other Yellowstone alkaline hot springs (Queens Laundry) (Meyer-Dombard et al., 2011) and, based on relative abundance, appears to prefer temperatures around 70°C although was present also at lower temperatures. The Yellowstone lineage is absent in the two Icelandic hot springs that we sampled, which, however, hosts a related archaeon, more closely related to *Candidatus* Caldiarchaeum subterraneum, over a similar temperature range (Supplementary Table S1, Figure S5). The other major group of Aigarchaeota was represented by several phylotypes related to the more recently described *Candidatus* Calditenuis aerorheumensis from Octopus Spring (Yellowstone), an organism proposed to potentially chemoautotrophically use oxygen as a terminal electron acceptor (Beam et al., 2016). Related lineages have been found in a variety of other alkaline and circumneutral hot spring including the Great Boiling Spring in Nevada (Rinke et al., 2013) Belcher Spring (Yellowstone) (Colman et al., 2015) Iceland (Mirete et al., 2011) and even in a shallow marine hydrothermal vent from Papua New Guinea (GenBank EF100632, Meyer-Dombard D.R., unpublished) (Supplementary Figure S5). In Mirror Pool these organisms inhabit the higher temperature range, unlike the Caldiarchaeum-type, presumably as the very low sulfide level allows sufficient dissolved oxygen. In the two Icelandic hot springs the Calditenuis-type lineages were present at much lower relative abundance. We could not test redox levels in the different niches of the hot spring runoff therefore these potential associations to oxygen and sulfide content remain speculative.

The dominant community members between 50–65°C were oxygenic and anoxygenic chlorophototrophs (diverse Cyanobacteria, Chloroflexi and *Chloracidobacterium*), forming characteristic green, orange and red mats depending on the site and temperature, as it has been shown in many other hot springs around the world (Miller et al., 2009; Klatt et al., 2013; Wang et al., 2013; Cuecas et al., 2014; Thiel et al., 2016). There were, however, some notable differences between Mirror Pool and the Icelandic features (Figure 6). *Synechococcus/Leptococcus*, an abundant member of the mats in Yellowstone, as well as *Gloeomargarita*, were absent in the two Icelandic hot springs. The striking absence of *Synechococcus* in Icelandic hot springs has been documented decades ago along with an overall lower diversity of thermophilic Cyanobacteria (Castenholz, 1969; Hreggvidsson et al., 2017). Two potential explanations have been proposed. As the light levels in Iceland severely decrease during the winter months, that could limit the survival of phototrophs that require a higher energy level. In addition, during the winter months at high latitudes, the temperature gradient changes, which would require upstream outflow colonization, a double challenge with the low light levels (Castenholz, 1969). Alternatively, it has been hypothesized that thermophilic *Synechococcus* and other species that are sensitive to freezing and desiccation are less likely to survive the time required for dispersal from sources in North America or Eurasia (Miller and Castenholz, 2000). As it has been well documented that some Cyanobacteria are highly sensitive to sulfide (Castenholz, 1977; Cohen et al., 1986; Skirnisdottir et al., 2000; Hamilton et al., 2012) that may also contribute to differences between the springs, as the levels in Vaðmálahver and Hurðarbak reach those toxic levels (∼1 mg/l). The Icelandic mats harbored, however, diverse other Cyanobacteria, primarily at 50°C and below, including *Chlorogloeopsis* and *Leptolyngbya*. We also observed important differences across Chloroflexi between the thermal regions. Chloroflexi are more tolerable to higher levels of sulfide and can detoxify it using a type II sulfide:quinone oxidoreductase or, some species, can use it as an electron donor for photoautotrophy (Bryant et al., 2012). The Mirror Pool mats are dominated by *Roseiflexus*, while the Icelandic mats are primarily composed of *Chloroflexus*. Mats with different abundance of *Chloroflexus* versus *Roseiflexus* have been found across hot springs in YNP and, because they were not correlated with the concentration of sulfide, unidentified environmental factors appear to drive those differences (Klatt et al., 2013). There are complex physiological interactions between photosynthetic autotrophs and heterotrophs in the mats as well as microbial partitioning driven by light levels, chemical gradients and competition (Ramsing et al., 2000; Ward and Cohan, 2005; Klatt et al., 2013; Cuecas et al., 2014; Nowack et al., 2015). Distinguishing the contribution of biogeography versus geochemistry in the composition of Icelandic versus Yellowstone mats clearly requires additional studies.

Above 70–73°C, the upper temperature limit for photosynthesis, the microbial mats disappeared sharply, and all communities were composed primarily of Archaea, Aquificae, Armatimonadetes and Thermi, as well as a variety of other phyla including uncultured lineages (Figure 5). As the highest temperature in Mirror Spring was 82°C, we could not directly compare strict hyperthermophilic communities (>85°C) between the Yellowstone and Iceland springs. Both sources of Hurðarbak and Vaðmálahver springs (90–100°C) were dominated by the strictly hyperthermophilic Crenarchaeota *Pyrobaculum* and *Ignisphaera* but also contained low levels of Nanoarchaeota. *Pyrobaculum* (ord. Thermoproteales) are predominantly anaerobes, with one species isolated form Iceland (*P. islandicus*) being a strict anaerobe, facultative lithoautotrophs (Huber et al., 2006). That may explain why they are abundant in the more reduced, sulfate rich and organic poor Hurðarbak and Vaðmálahver. *Ignisphaera*, an anaerobic heterotroph, a less characterized member of the Desulfurococcales, with one species isolated from New Zealand being very sensitive to sodium chloride (Niederberger et al., 2006) which may explain its absence from Mirror Pool. Hurðarbak also had a significant population of *Thermocrinis*, an autotrophic member of the Aquificae that dominated the community at 80 °C but was a minor component in both Vaðmálahver and Mirror Pool. As *Thermocrinis* have been isolated both from YNP and Iceland (Huber et al., 1998; Eder and Huber, 2002), it is not clear what causes their differential relative abundance between the springs we studied. Another chemical characteristic that we cannot clearly link to a microbial differential feature is the significantly more abundant organic carbon in Mirror Pool, although that may result in overall higher microbial productivity. The relatively few studies that have correlated chemical gradients of springs with microbial composition and productivity revealed limiting energetic and nutritional factors that impact the communities in different microenvironments (Macur et al., 2004; Colman et al., 2016; Lindsay et al., 2018; Havig and Hamilton, 2019). Future combined analyses of datasets generated from multiple studies and locations should better define environmental parameters that shape the composition of those communities and the abundance of specific organisms.

### Potential Endemism at Icelandic and Yellowstone Springs

**FIGURE 7:**
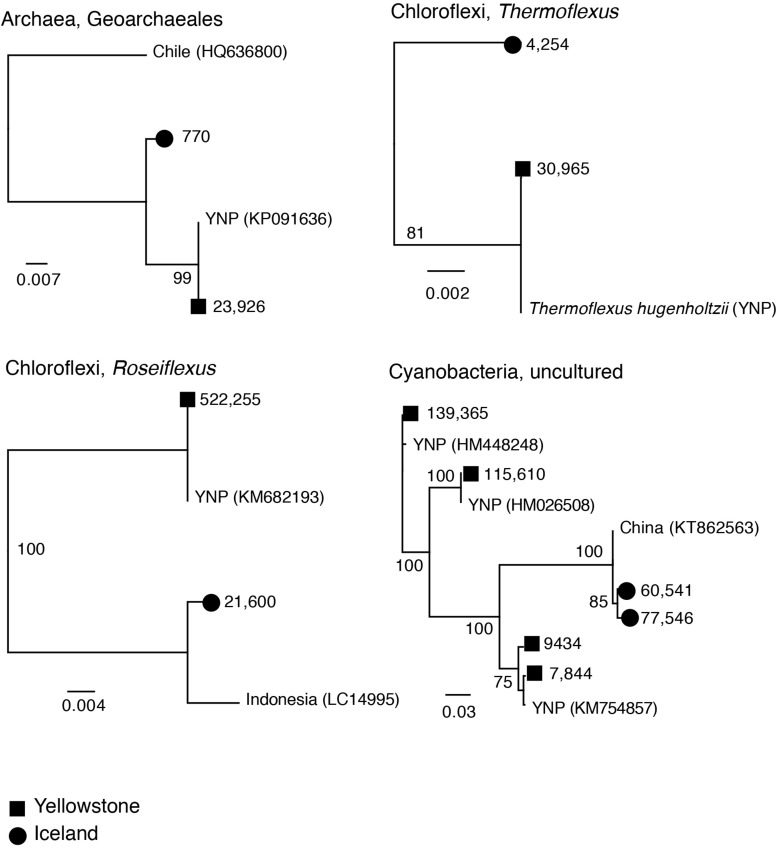
Neighbor joining trees (JC corrected distances) of potential endemic amplicon sequence variants (ASVs) from Iceland and Yelowstone (Mirror Pool). Numbers on branch tips indicate abundance across the entire dataset. Reference sequences and accession numbers from public rRNA databases and location were included. The numbers at nodes indicate bootstrap support. Scale bar indicates inferred number of substitutions per site.

Inspection of unique sequence variants revealed potential lineages that may be endemic to hot springs in Iceland or Yellowstone. Geographic separation of thermophilic communities or Archaea and Cyanobacteria between multiple sites across three continents has previously been shown to result in divergence of local populations that would ultimately lead to speciation (Castenholz, 1969; Papke et al., 2003; Whitaker et al., 2003). Therefore, phylogenetic analysis was used to characterize several closely related sequences between Icelandic and Yellowstone hot springs, to test whether short amplicon sequences many provide sufficient resolution. The included taxa were Archaea, Chloroflexi and Cyanobacteria lineages (Figure 7), taxa that have previously analyzed in the context of biogeography. The genetic distance between those sequences suggests indeed they represent related but distinct species or subspecies. Some of the sequences from Mirror Pool are very closely related or identical to prior sequences reported from other Yellowstone springs. Because we did not find close homologs from Icelandic hot springs in rRNA sequence databases, the analyzed Vaðmálahver and Hurðarbak amplicons had their closest relatives in hot springs from China. The analysis of the Aigarchaeota phylotypes (Supplementary Figure S5), discussed in the previous section, may also point to endemism, potentially driven by a combination of environmental factors (temperature and redox levels) and geographic separation. Across the entire dataset, a large fraction of ASVs were specific to either Yellowstone or Iceland. Specifically, out of the 654 ASVs with at least 100 sequences between all samples (>0.003% of the 3.8 million sequences assigned to ASVs), 56% were only identified in the Icelandic springs, 37% only in Yellowstone, while only 7% of the ASVs were common between YNP and Iceland. Some of unique ASVs were high abundance taxa (e.g., a *Ca.* Caldiarchaeum ASV was represented by over 50,000 sequences from Iceland and no sequence from YNP while a *Chloroflexus* ASV had ∼240,000 sequences from YNP and no sequence from Iceland). The distribution of all ASVs in the Icelandic and Yellowstone springs and their taxonomic classification is provided in the Supplementary Table S2. Because the resolution enabled by ASVs varies across microbial taxa, it is not possible to conclude that certain species or subspecies are unique to one of another location, as multiple ASVs were classified to the same taxonomic rank.

While preliminary, based on few sampled sites and short sequences, these observations support the hypothesis that endemic lineages evolved in those hot springs and are in line with the previous studies comparing Icelandic geothermal systems with those from North America, continental Europe and Asia. The taxon differences could also be due to differences in chemical composition of the hot springs, which may have selected for related but globally distributed species. Nevertheless, the studies by Papke and colleagues (Papke et al., 2003; Papke and Ward, 2004) that included more locations and deeper phylogenetic analyses, concluded that chemical differences did not explain observed differences in species distribution across continents. Independent analyses of hot springs across the world should therefore enrich our understanding of the links between the ecological diversity and evolutionary history of thermophilic organisms, at local and global scales.

## Author’s Note

This manuscript has been released as a pre-print at bioRxiv (https://doi.org/10.1101/841700), Podar P.T. et al., “Comparative analysis of microbial diversity across temperature gradients in hot springs from Yellowstone and Iceland”, Dec. 11, 2019 (Podar et al., 2019).

## Data Availability Statement

The raw FASTQ files are available in the NCBI SRA (accession numbers SRR11066910–SRR11066942, Bioproject PRJNA605860).

## Author Contributions

PP and MP designed the study analyzed the data and wrote the manuscript with input from SB and ZY. PP, SB, and MP collected the environmental samples. PP and ZY performed the DNA extraction, amplification, and sequencing. All authors contributed to the article and approved the submitted version.

## Conflict of Interest

The authors declare that the research was conducted in the absence of any commercial or financial relationships that could be construed as a potential conflict of interest.

## Footnotes

1 www.geneious.com

2 http://rcn.montana.edu/Features/Detail.aspx?id=8999
