# Supplementary figures and images for: Comparative Analysis of Microbial Diversity Across Temperature Gradients in Hot Springs From Yellowstone and Iceland
Source: Front Microbiol. 2020 Jul 14;11:1625. doi: 10.3389/fmicb.2020.01625 (PMC7372906; doi:10.3389/fmicb.2020.01625)

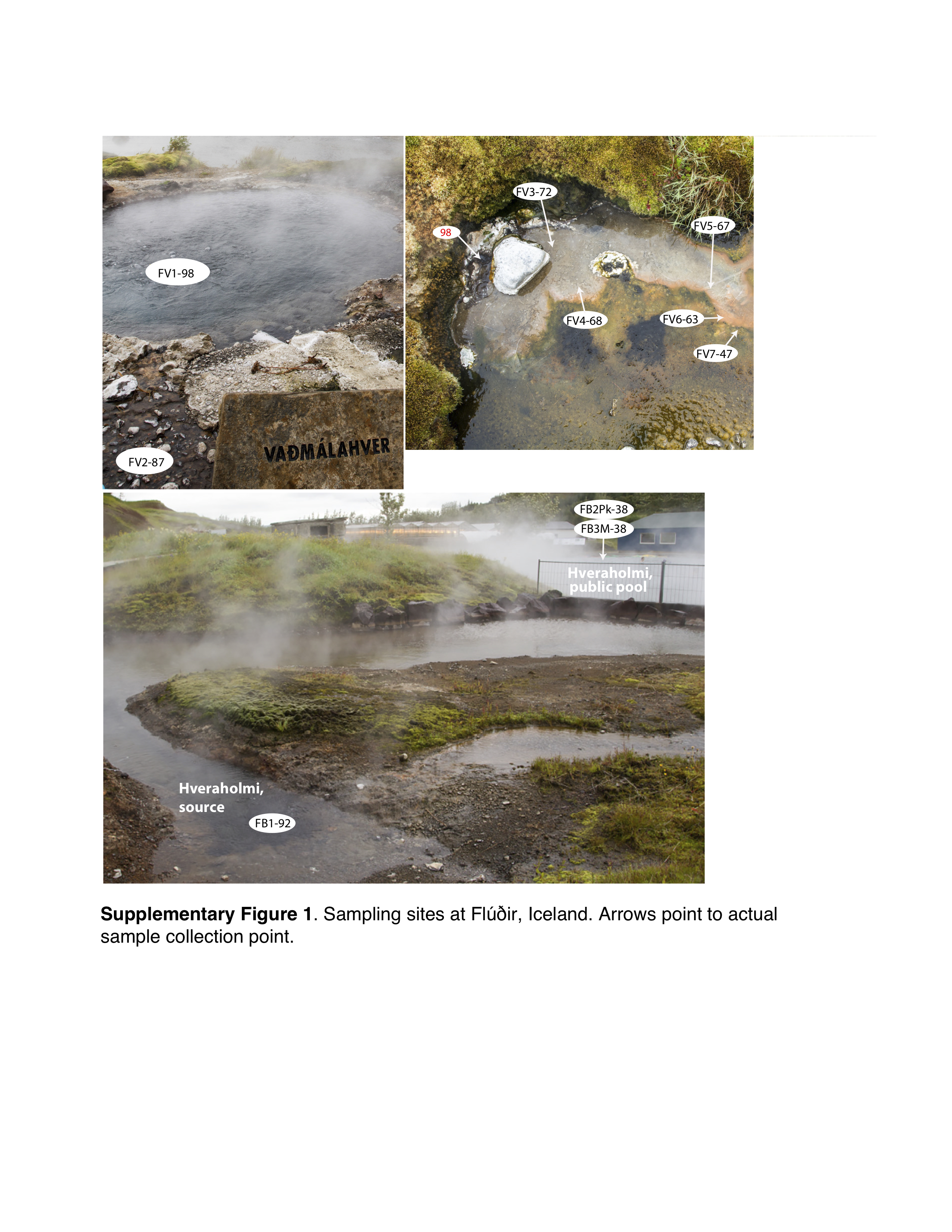

Supplement: Supplementary file 1 [file Image_1.JPEG]

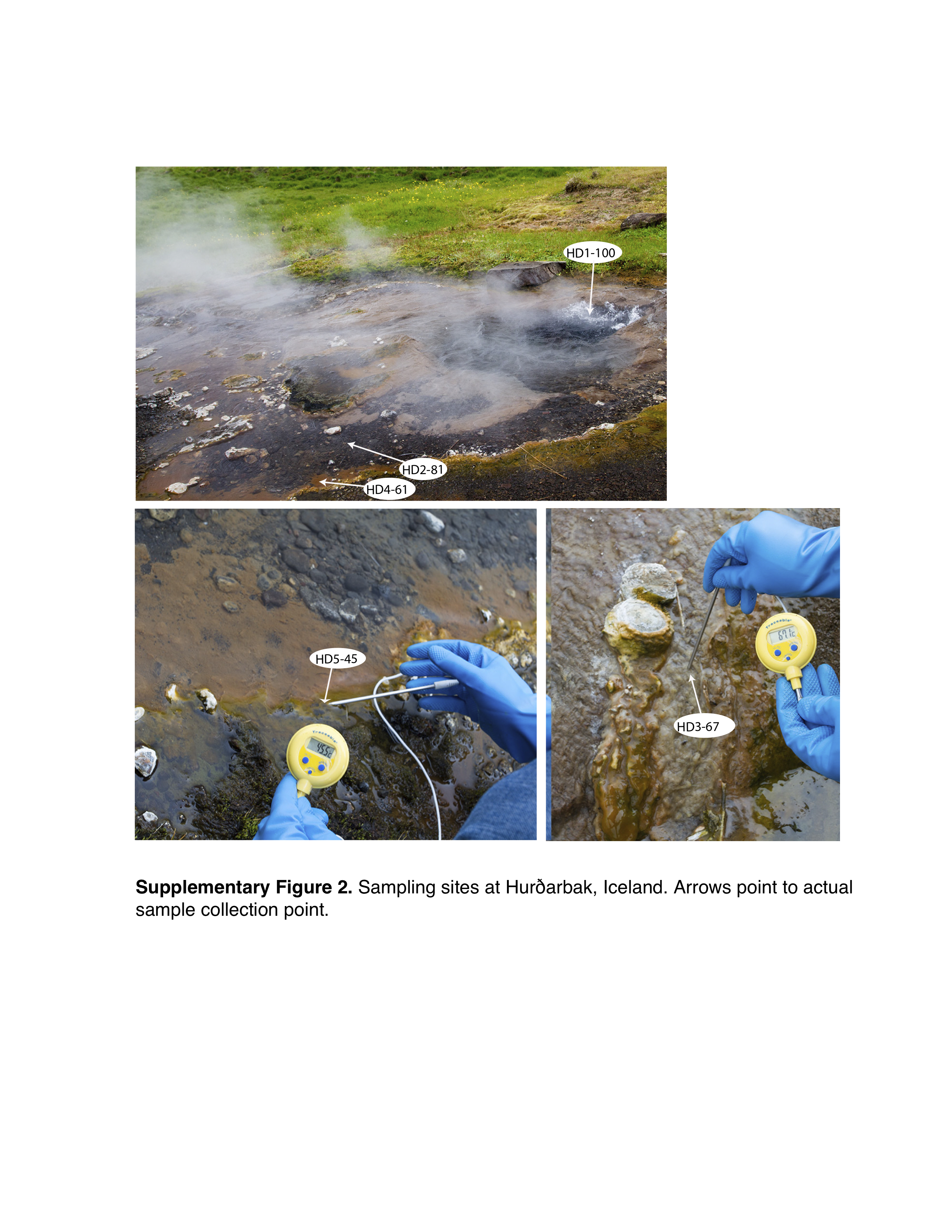

Supplement: Supplementary file 2 [file Image_2.JPEG]

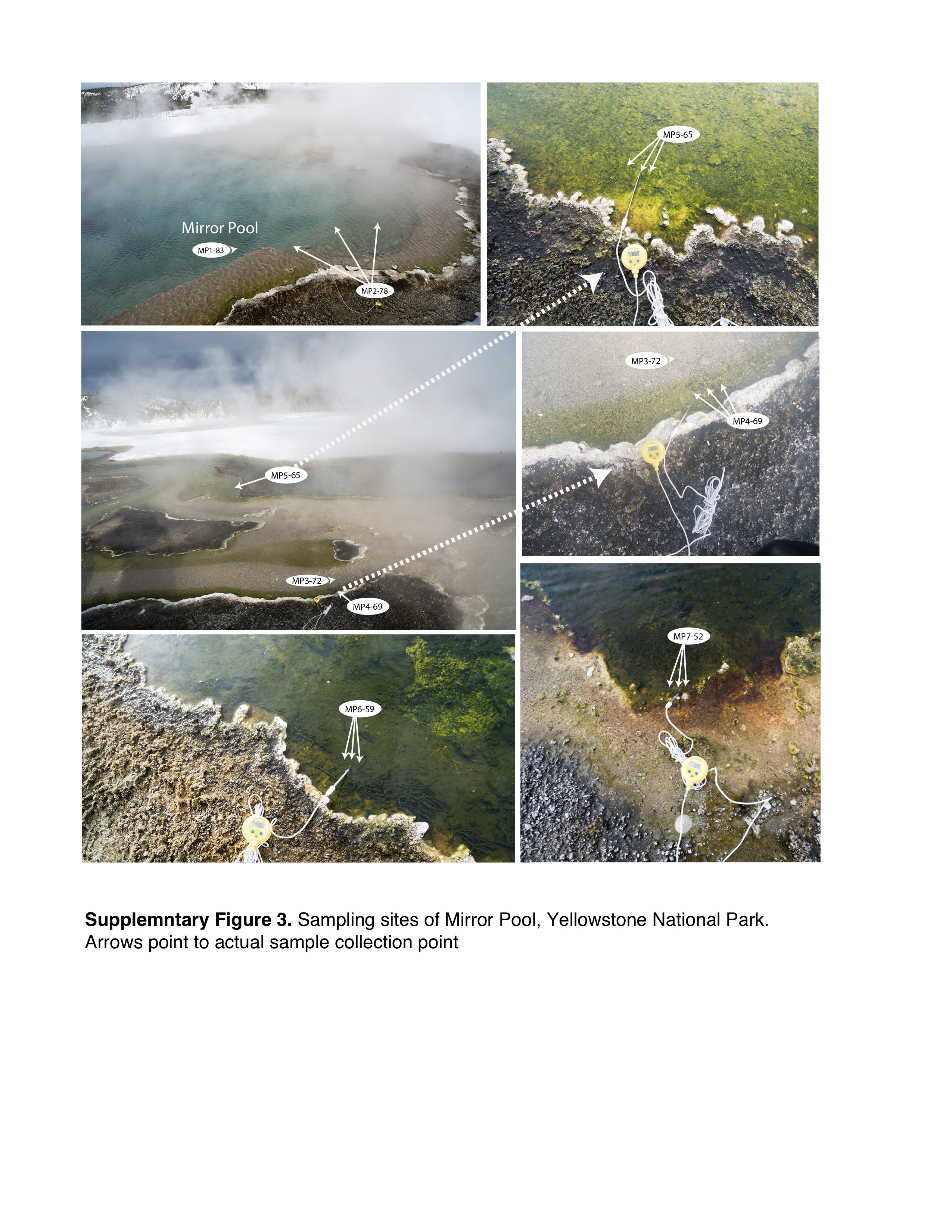

Supplement: Supplementary file 3 [file Image_3.JPEG]

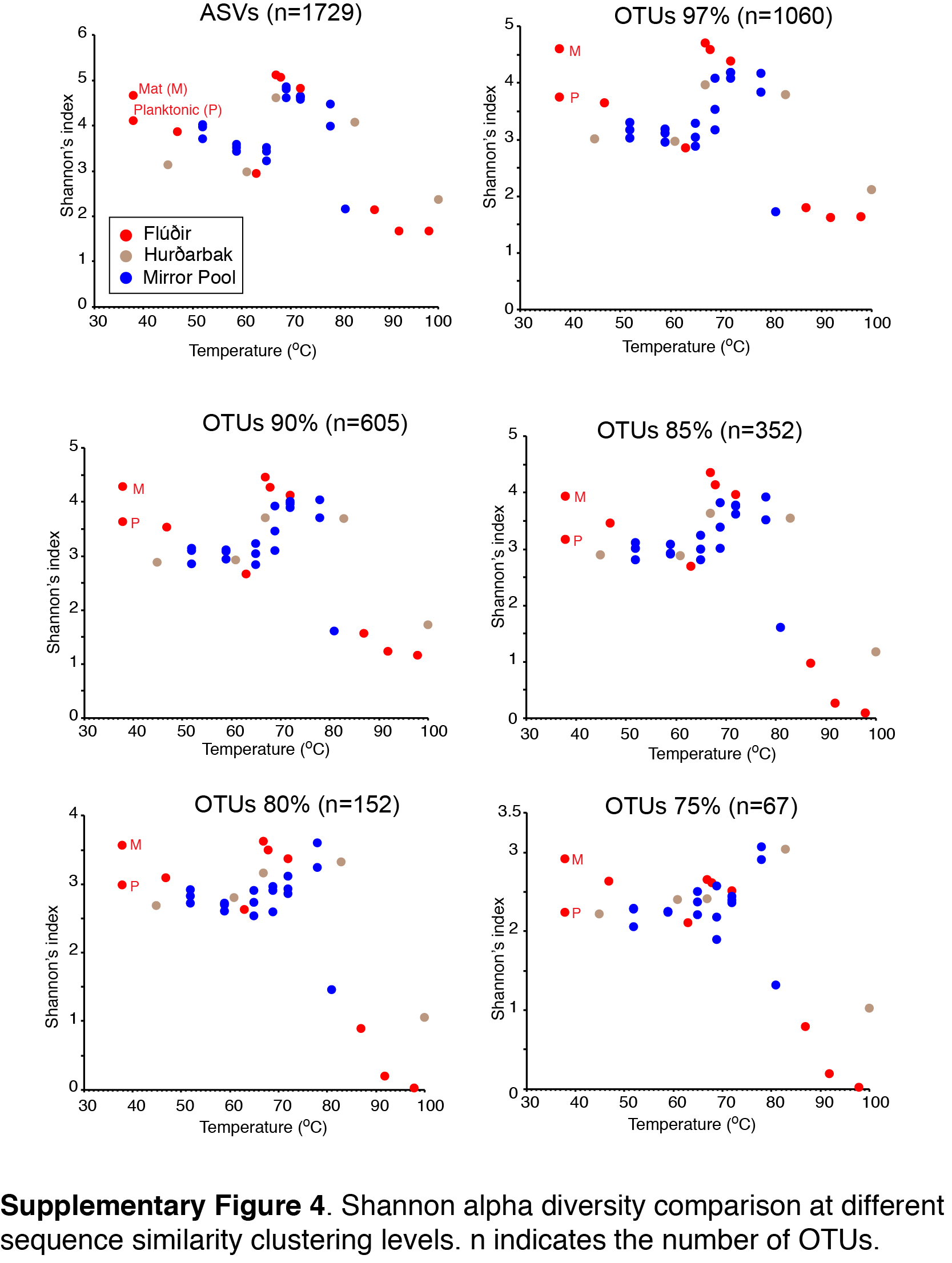

Supplement: Supplementary file 4 [file Image_4.JPEG]

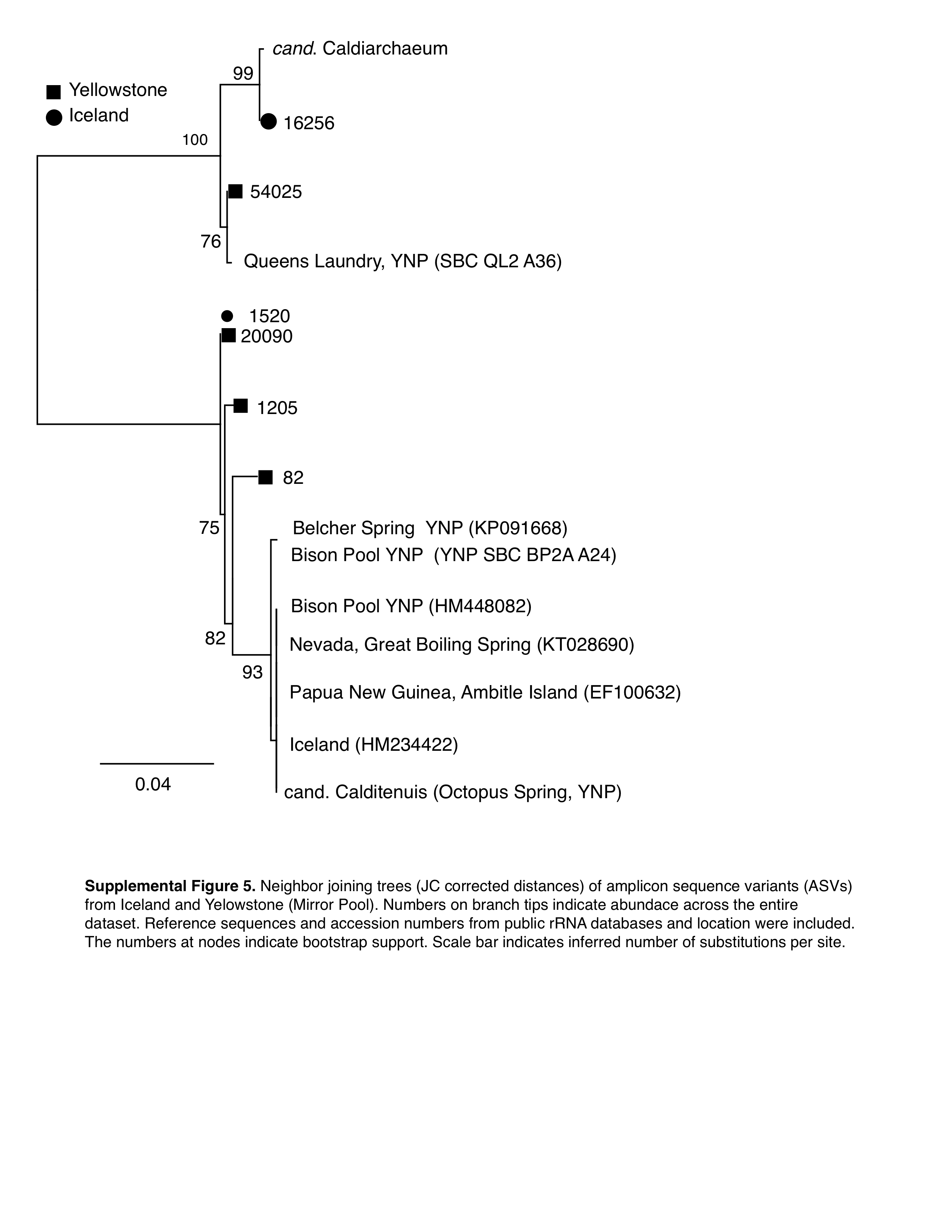

Supplement: Supplementary file 5 [file Image_5.JPEG]
