# Supplementary material for: Comparative Analysis of Microbial Diversity Across Temperature Gradients in Hot Springs From Yellowstone and Iceland
Source: Front Microbiol. 2020 Jul 14;11:1625. doi: 10.3389/fmicb.2020.01625 (PMC7372906; doi:10.3389/fmicb.2020.01625)
Supplement: Supplementary file 6 [file Table_1.DOCX]

| SampleID | Location | Thermal Feature | Temperature, ^o^C | Replicate |
| --- | --- | --- | --- | --- |
| MP1A | YNP | MirrorPool | 52 | 1 |
| MP1B | YNP | MirrorPool | 52 | 2 |
| MP1C | YNP | MirrorPool | 52 | 3 |
| MP2A | YNP | MirrorPool | 58.6 | 1 |
| MP2B | YNP | MirrorPool | 58.6 | 2 |
| MP2C | YNP | MirrorPool | 58.6 | 3 |
| MP3A | YNP | MirrorPool | 65.5 | 1 |
| MP3B | YNP | MirrorPool | 65.5 | 2 |
| MP3C | YNP | MirrorPool | 65.5 | 3 |
| MP4A | YNP | MirrorPool | 69 | 1 |
| MP4B | YNP | MirrorPool | 69 | 2 |
| MP4C | YNP | MirrorPool | 69 | 3 |
| MP5A | YNP | MirrorPool | 72.5 | 1 |
| MP5B | YNP | MirrorPool | 72.5 | 2 |
| MP5C | YNP | MirrorPool | 72.5 | 3 |
| MP6A | YNP | MirrorPool | 78 | 1 |
| MP6B* | YNP | MirrorPool | 78 | 2 |
| MP6C | YNP | MirrorPool | 78 | 3 |
| MP6D | YNP | MirrorPool | 83 | 1 |
| FV2 | Iceland | Vaðmálahver | 87 | 1 |
| FB1 | Iceland | Hverahólmi sr. | 92 | 1 |
| FB2.Pk | Iceland | Hverahólmi | 38 | 1 |
| FB3.Mat | Iceland | Hverahólmi | 38 | 1 |
| FV1 | Iceland | Vaðmálahver | 98 | 1 |
| FV3 | Iceland | Vaðmálahver | 72 | 1 |
| FV4 | Iceland | Vaðmálahver | 68 | 1 |
| FV5 | Iceland | Vaðmálahver | 67 | 1 |
| FV6 | Iceland | Vaðmálahver | 63 | 1 |
| FV7 | Iceland | Vaðmálahver | 47 | 1 |
| HD1 | Iceland | Hurðarbak | 100 | 1 |
| HD2 | Iceland | Hurðarbak | 81 | 1 |
| HD3 | Iceland | Hurðarbak | 67 | 1 |
| HD4 | Iceland | Hurðarbak | 61.4 | 1 |
| HD5 | Iceland | Hurðarbak | 45.5 | 1 |

Table 1. Environmental samples used in the study. Except for FB2.Pk, which

was a water sample, all others were mats/sediment. MP6B* did not yield

sufficient sequences.
