# Supplementary material for: Comparative Analysis of Microbial Diversity Across Temperature Gradients in Hot Springs From Yellowstone and Iceland
Source: Front Microbiol. 2020 Jul 14;11:1625. doi: 10.3389/fmicb.2020.01625 (PMC7372906; doi:10.3389/fmicb.2020.01625)
Supplement: Supplementary file 7 [file Table_2.DOCX]

|  | Mirror Pool | Vaðmálahver | Hurðarbak |
| --- | --- | --- | --- |
| TC | 101.6 | 24.39 | 11.94 |
| TIC | 4.84 | 3.01 | 2.24 |
| TOC | 96.79 | 21.38 | 9.71 |
| Cl | 271.79 | 24.89 | 32.01 |
| NO3 | 0.19 | 0.06 | 0.07 |
| SO4 | 13.78 | 54.97 | 57.13 |
| HPO4 | 0.1 | 0.13 | 0.15 |
| F | 19.34 | 1.14 | 1.84 |
| Na | 400.09 | 78.23 | 69.87 |
| K | 10.3 | 0.73 | 0.83 |
| Mg | 0.02 | 0.11 | 0.08 |
| Ca | 0.75 | 5.89 | 4.26 |
| Al | 0.33 | 0.05 | 0.05 |
| Cu | 0 | 0 | 0.01 |
| Fe | 0.04 | 0.15 | 0.17 |
| Si | 119.55 | 31.38 | 20.23 |
| S | 4.69 | 25.03 | 23.01 |
| Ni | 0.03 | 0.01 | 0 |
| Pb | 0.18 | 0.04 | 0.03 |
| Cr | 0.01 | 0.01 | 0.01 |
| Be | 0.01 | 0 | 0 |
| As | 1.32 | 0.01 | 0.02 |
| Sb | 0.03 | 0 | 0 |
| Y | 0.01 | 0.01 | 0.01 |

**Table 2**. Chemical composition of hot spring water samples.

All values in mg/l. TC, total carbon; TIC, total inorganic carbon; TOC, total organic carbon. Mn, Zn, Cd, Co, Ba, Se, Tl, Ag: not detected (<0.01 mg/l).
